# Pulmonary toxicity of immune checkpoint immunotherapy

**DOI:** 10.1172/JCI170503

**Published:** 2024-01-16

**Authors:** Mohammad I. Ghanbar, Karthik Suresh

**Affiliations:** 1 Division of Pulmonary & Critical Care Medicine, Department of Medicine, and; 2 Department of Oncology, Johns Hopkins University School of Medicine, Baltimore, Maryland, USA.

## Abstract

Cancer remains a leading cause of mortality on a global scale. Lung cancer, specifically non–small cell lung cancer (NSCLC), is a prominent contributor to this burden. The management of NSCLC has advanced substantially in recent years, with immunotherapeutic agents, such as immune checkpoint inhibitors (ICIs), leading to improved patient outcomes. Although generally well tolerated, the administration of ICIs can result in unique side effects known as immune-related adverse events (irAEs). The occurrence of irAEs involving the lungs, specifically checkpoint inhibitor pneumonitis (CIP), can have a profound effect on both future therapy options and overall survival. Despite CIP being one of the more common serious irAEs, limited treatment options are currently available, in part due to a lack of understanding of the underlying mechanisms involved in its development. In this Review, we aim to provide an overview of the epidemiology and clinical characteristics of CIP, followed by an examination of the emerging literature on the pathobiology of this condition.

## Introduction

The relationship between the immune system and cancer growth is complex; the interplay between the immune system and the tumor impacts all aspects of cancer growth, from tumorigenesis to tumor growth and eventual metastasis. Such interaction between the immune system and the tumor is based on the theory of cancer immunoediting, which involves 3 phases: elimination, equilibrium and escape (1). In the elimination phase, a competent immune system attacks and destroys tumor cells. Tumor cells that survive the initial attack may enter a state of dormancy known as the equilibrium phase before entering the escape phase, in which tumor cells acquire the ability to evade the immune system and grow unchecked (1). Tumors can escape immune surveillance through various mechanisms, including alteration or loss of antigens, upregulation of immune checkpoint molecules, and manipulation of cytokines and oncogenic signaling to create an immune-suppressive tumor microenvironment (2, 3). Attempts to reengage the immune system to counteract cancer-induced immune evasion have resulted in the emergence of various immunotherapeutic modalities (4, 5). Immune checkpoint inhibitors (ICIs) are one such immunotherapeutic intervention.

Immune checkpoints are a group of proteins that are expressed on the surface of various cells, and they play a crucial role in modulating immune responses. However, immune checkpoints have been exploited by cancer cells for immune evasion (6). In turn, ICI therapy seeks to overcome this unfavorable immune-cancer interaction. ICIs have been approved to treat several types of cancer, including melanoma, lymphoma, renal cell, and lung cancer. Particularly, ICIs are increasingly being used as a first-line option for both early and advanced stages of non–small cell lung cancer (NSCLC) (7), a leading cause of cancer-related deaths globally (8). The use of ICIs has shown improved overall survival in NSCLC (9), but it has also been linked to immune-related adverse events (irAEs), including lung toxicity (10, 11). Checkpoint inhibitor pneumonitis (CIP) is a major irAE associated with significant morbidity and mortality (12, 13). Despite this clinical significance, the mechanisms underlying lung injury in CIP are not well understood, limiting the availability of specific treatments. In this Review, we outline the mechanisms of action and use of ICIs, the clinical features of CIP, and recent research aimed at understanding the biological underpinnings of this condition.

## ICI biology

T cells are an integral part of the adaptive immune response; their activation by an offending antigen (e.g., an infectious agent or a tumor antigen) can propagate a series of inflammatory responses. In cancer, these antigens can arise from tumor cells; as such, T cells are known to be an important factor in immune cancer surveillance. However, T cell activation is naturally balanced by counterregulatory mechanisms in the form of immune checkpoints, which serve to curb the immune response and avoid autoimmunity (14). This natural phenomenon in turn is exploited by cancer cells to induce immune tolerance.

The natural T cell–mediated immune response is a complex process that involves multiple steps and interactions with other cell types aimed at targeting specific foreign antigens or epitopes. During immune surveillance, antigen-presenting cells (APCs) play a crucial role in this process by recognizing tumor antigens and activating T cells (1). T cell activation requires two signals: a primary signal transmitted through its T cell receptor and a costimulatory signal delivered by the CD28 receptor. This costimulation occurs when APC surface proteins B7-1 (CD80) or B7-2 (CD86) interact with CD28 (6). This costimulatory signal is essential for proper T cell activation and function.

**Figure 1:**
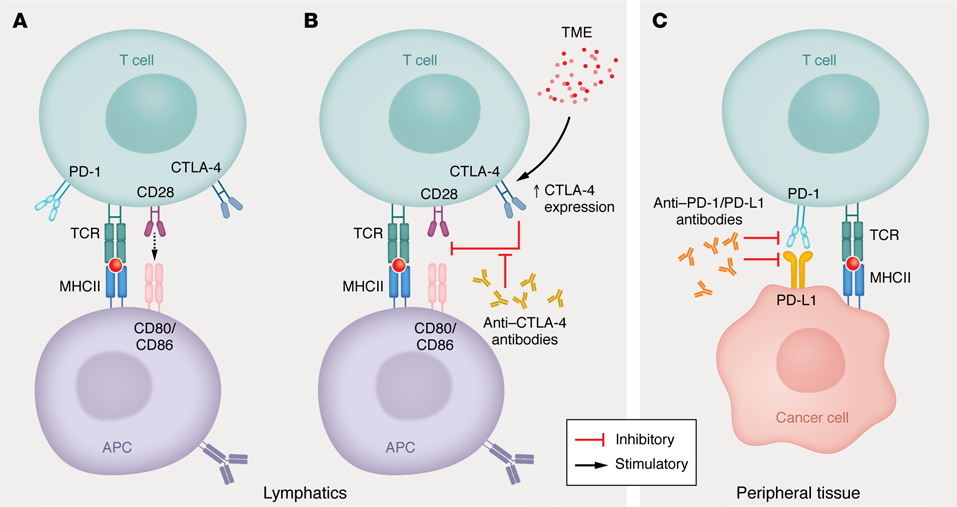
Immune checkpoint biology. (**A**) T cell activation is initiated by interacting via its T cell receptor with APCs that present offending antigens. A costimulatory signal is additionally required on the T cell CD28 receptor for full activation. Upon activation, CTLA-4 and PD-1 are expressed on the cell surface. (**B**) CD28 costimulation can be inhibited by CTLA-4, leading to T cell dysfunction. CTLA-4 expression can be enhanced by the tumor microenvironment (TME). Both of these steps occur at the level of the lymphoid organs. (**C**) Stimulation of the PD-1 receptor by tumor-expressed PD-L1 can render activated T cells dormant, inhibiting immune responses against cancer cells. These mechanisms can be overcome with specific antibodies against the receptors (CTLA-4, PD-1) or ligands (PD-L1).

Cytotoxic T lymphocyte–associated protein-4 (CTLA-4) and programmed cell death protein-1 (PD-1) receptors are critical checkpoints that function at distinct stages of the T cell activation cycle. CTLA-4 competes with CD28 for their shared ligands, CD80 or CD86 (15), limiting further T cell activation (16). This process occurs at the start of the T cell activation cycle. T cells subsequently can become unresponsive or undergo a state of anergy (17). At the same time, CTLA-4 signaling in T cells promotes their transformation into Tregs (18) that are essential in peripheral immune tolerance (19). CTLA-4 expression in lymphocytes is thought to be driven by lung cancer or the tumor microenvironment (20). While CTLA-4 regulates the early process of T cell activation within the lymph nodes, PD-1 checkpoints function at a later stage in the peripheral tissues. In contrast to that of CTLA-4, PD-1 expression is observed at the late effector phase of the activated T cell (21). Upon its interaction with programmed cell death ligand-1 or -2 (PD-L1 or PD-L2), intracellular pathways are triggered that inhibit T cell receptor signaling, decrease proliferation, and suppress effector functions (22). PD-1’s ligands are expressed on a wide variety of nonhematopoietic cells, including endothelial and epithelial cells (23). Cancer cells exploit the inhibitory function of the PD-1 checkpoint pathway by overexpressing PD-L1 or PD-L2, thereby limiting the host’s immune response (24) (Figure 1).

In addition to the CTLA-4 and PD-1 pathways, lymphocyte activation gene 3 (LAG-3) is a known checkpoint involved in immune regulation. LAG-3 is primarily found on lymphocytes, but it can also be expressed in NK and myeloid cells. LAG-3 activation by interaction with its primary ligand, the major histocompatibility II receptor found on APCs, can impede T cell proliferation and differentiation (25). In vivo pharmacological blockade or genetic ablation of LAG-3 has been consequently shown to restore function in exhausted T cells, leading to an enhanced antitumor response (26). However, simultaneous inhibition of LAG-3 and PD-1 function resulted in the optimal antitumor response in murine models (27). Therefore, clinically, the use of LAG-3 blocking antibodies has only been approved in combination with PD-1 blockade (28), and limited data are available on its pulmonary toxicity profile. Other checkpoint proteins, including B and T cell lymphocyte attenuator (BTLA), V-domain Ig suppressor of T cell activation (VISTA), and T cell immunoglobulin and mucin domain 3 (TIM-3) are also being investigated as potential targets for inhibition in early-stage clinical trials (29). Like PD-1 and LAG-3, these proteins all play a role in limiting T cell activity and effector function.

While clearly important in cancer biology, these pathways also play a pivotal role in maintaining immune homeostasis. This is best evidenced by the emergence of spontaneous autoimmunity in CTLA-4– and PD-1–knockout murine models (14, 30, 31). It is noteworthy to mention that the loss of CTLA-4 inhibition in murine models leads to fatal disease soon after birth, in contrast to PD-1–knockout mice that develop nonlethal autoimmunity (30, 32). Unsurprisingly, disruption of these pathways through therapeutic blockade, while beneficial for tumor control, also incurs the risk of autoimmune dysfunction. Such dysfunction clinically manifests as irAEs in patients with cancer undergoing ICI treatment. irAEs can involve any organ, frequently affecting the skin as dermatitis, the gastrointestinal tract as colitis, and the endocrine system in the form of thyroiditis or hypophysitis. Of particular importance to this Review, lung involvement can present in several ways, most often as pneumonitis (33).

## Pulmonary irAEs

ICI therapy has been linked to several complications that affect the lungs. These include sarcoid-like granulomatosis (34–37), pleural effusion (38), exacerbation of obstructive lung disease (37, 39–43), and, most notably, CIP. Among these complications, CIP is the most widely recognized and is of serious concern due to its high morbidity and mortality rates (12, 44). In fact, CIP is the leading cause of fatal irAEs in patients receiving anti–PD-1/PD-L1 monotherapy (13).

At first, CIP incidence was thought to be low; CIP incidence in clinical trials was reported to be <6% (38, 45–49). However, this assertion has been challenged by real-world data, which revealed higher rates ranging between 10% and 20% (44, 50, 51). Various factors have been shown to increase the risk of CIP, including use of PD-1/PD-L1 agents (52, 53) (compared with CTLA-4 inhibitors), combination immunotherapy (54, 55) (CTLA-4 and PD-1 combination rather than monotherapy), radiotherapy (56, 57), and the organ of tumor origin (58, 59) (e.g., NSCLC vs. renal cell or other cancer types). More recently, the incorporation of ICIs into neoadjuvant therapy for resectable lung cancer has revealed similar rates of CIP to those witnessed with adjuvant treatment (ranging from 1.1% to 6.4%, see Table 1). However, these findings necessitate careful interpretation for several reasons. First, it’s crucial to acknowledge that pneumonitis rates primarily stem from phase II clinical trials, which typically involve a smaller participant pool that may not reflect the general population. Second, our experience from the adjuvant trials underscores a concern that the reported rates of pneumonitis in these controlled trial settings may underestimate the true incidence observed in real-world clinical practice. Additionally, it’s noteworthy that a significant subset of participants receive radiotherapy, a risk factor linked with elevated pneumonitis incidence. Moreover, the integration of both neoadjuvant and adjuvant treatments in certain trial protocols further complicates the overall assessment of pneumonitis risk. A summary of the trials and their reported pneumonitis rates is provided in Table 1.

The clinical presentation of CIP includes dyspnea, cough, and hypoxemia (either at rest or with exertion) (60). These symptoms may occur soon after treatment initiation, typically within days to weeks (61). The time frames reported vary among study cohorts; however, the reported median onset is approximately 2–3 months after ICI initiation (12, 51, 61). The common terminology criteria for adverse events (62) can be utilized to grade the overall clinical severity of CIP, as outlined in Table 2.

Pathognomonic radiological features for CIP do not exist. Instead, a range of changes have been identified on CT scans, with approximately 50% of the cases reported in the literature presenting either as ground-glass opacities or consolidative lesions (63). The diagnosis of CIP is established after excluding other potential etiologies, such as infection, tumor progression, and alveolar hemorrhage. Although bronchoalveolar lavage fluid (BALF) may be employed to exclude alternative diagnoses or infections, it is variably employed and is not considered an essential diagnostic procedure (64).

Once a diagnosis of CIP is established, ICI therapy is typically terminated, and systemic glucocorticoid therapy is initiated for the majority of patients with more severe (>grade 1) disease. However, a subset of patients fails to respond to steroids and may require additional immunosuppressive interventions beyond glucocorticoids (61, 65, 66). Subsequent rechallenge with ICIs after CIP resolution is typically possible in only a small percentage of patients, thereby limiting future immunotherapy for a substantial proportion of affected individuals (12, 67).

## Pathophysiology of CIP

The clinical presentation of CIP is similar to that of acute lung injury. However, despite being a well-recognized irAE, the pathophysiology of CIP, similar to other irAEs, remains largely elusive. Nevertheless, preliminary findings suggest the involvement of several mechanistic pathways in the development of CIP, which are described below.

### Cellular autoimmunity/higher T cell activity.

**Figure 2:**
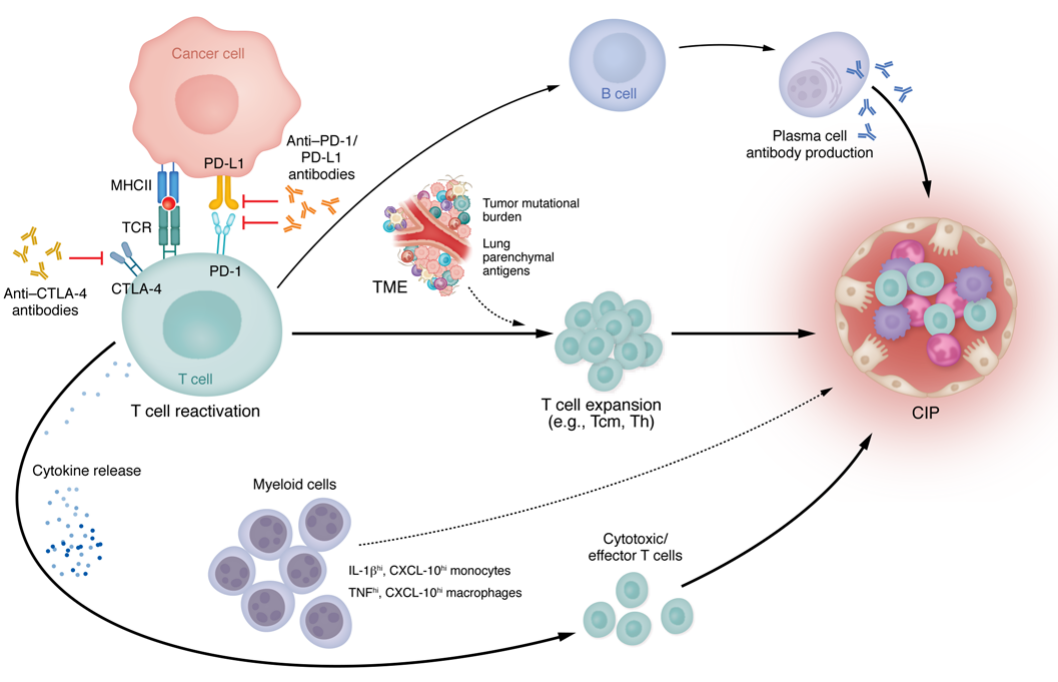
Pathophysiological mechanisms in CIP. The use of ICI liberates T cells from cancer-induced immunosuppression. This also triggers a number of pathways that include B and plasma cell proliferation and subsequent autoimmune antibody production (e.g., anti-CD74); release of cytokines (e.g., IL-1β, TNF-α, CXCL-10) that are involved in inflammation and can affect multiple cell types and expansion of T cells (e.g., Tcm, Th, clonal T cells) that are likely influenced by the tumor microenvironment (TME), tumor mutational burden, and self-antigens in the lung parenchyma. These different pathways converge individually or in combination to cause inflammatory damage in the lung leading to CIP. The involvement of myeloid cells in CIP is evident although not well defined. They either may act as an additional stimulus for T cell activation and expansion or are regulated by the T cell and cytokine milieu, contributing to pulmonary injury. Solid lines indicate known mechanisms involved in CIP; dashed lines indicate proposed mechanisms.

There is accumulating evidence to suggest that T cell upregulation may be involved in the pathogenesis of CIP (Figure 2). Many investigators have shown an increased number of overall T cells in addition to certain T cell subsets. Notably, in our study, the BALF of patients with CIP has shown a significant increase in CD4^+^ T cells (68). In 12 patients with CIP, CD4^+^CD45RA^–^CD62L^+^ central memory T (Tcm) cells were found to be increased (68). Tcm cells are derived from either CD4^+^ or CD8^+^ lymphocytes that circulate in the blood and target secondary lymphoid tissues to quickly propagate in response to familiar antigens (69), leading to a more rapid and augmented immune response (70). Additionally, Tcm cells have previously been shown to be resistant to steroid-induced apoptosis (71), which may account for the steroid-refractory nature of CIP in some patients. Moreover, CD62L has been demonstrated to facilitate the migration of T cells to sites of inflammation (72). This notion receives further support from research conducted on peripheral blood T cell profiling in patients with melanoma who underwent ICI treatment. Among the 18 individuals studied who later suffered irAEs, those with higher pretreatment levels of circulating Tcm cells were found to develop severe irAEs (73). In another study, researchers examined 11 patients with CIP by performing BALF sampling and conducting single-cell RNA and T cell sequencing. The transcriptomic signature of these patients showed an accumulation of Th lymphocytes, specifically Th17.1 (74), a subset of Th 17 cells that produce IFN-γ and are implicated in a number of autoimmune diseases (75). Among these Th17.1 lymphocytes, a unique cluster was identified that had a distinct transcriptomic signature characterized by genes related to cytotoxicity and monocyte activation. Using trajectory inference, they demonstrated that the Th17.1 lymphocyte colony in CIP BALF samples has plasticity and undergoes pathogenic skewing toward this IFN-γ and monocyte activation phenotype. At baseline, Th17 cells play a pivotal role in upholding gut barrier defenses, facilitating granulocyte maturation and chemotaxis, and contributing to immunity against extracellular pathogens (76). The depletion of proinflammatory Th17 cells heightens vulnerability to infections caused by *Staphylococcus aureus* and *Candida albicans*, culminating in recurrent skin and pulmonary infections (77). Concurrently, an excessive Th17 cell response can precipitate autoimmune reactions. In particular, Th17.1 lymphocytes have been implicated in driving neutrophilic inflammation, granuloma formation, and provoking resistance to corticosteroid treatment (75). Elevated quantities of these cells have been demonstrated in BALF from individuals with sarcoidosis, and they are associated with active lung disease (78–80). It is conceivable that the equilibrium of such a cell population may be disrupted in the context of ICI therapy, as demonstrated in a murine model in which checkpoint inhibition triggered the activation of Th17 lymphocytes (81).

While these findings support the hypothesis that proinflammatory T cell subsets contribute to alveolar damage in CIP, other studies have suggested that the proliferating T cells may be clonal. For example, investigators have identified identical T cell clones in tumor tissue and the site of irAEs, albeit in extrapulmonary locations. Autopsy specimens from two patients with melanoma who died from myocarditis after receiving combination therapy with CTLA-4/PD-1 antibodies revealed shared T cell clones in the tumor, heart, and skeletal muscle, without any evidence of adjacent tissue involvement, including the smooth muscle (82). Similarly, of 73 patients with NSCLC who received anti–PD-1 agents, 25 developed skin toxic effects, and nine shared T cell antigens that were detected in both cancer and skin tissue (83). A potentially similar mechanism may also exist in CIP. Analysis of T cell receptor sequencing data from four patients who had developed pneumonitis while undergoing PD-1 blockade therapy revealed the presence of overlapping T cell clones in both the lung and tumor tissue (84). These clones were absent in peripheral blood and secondary lymphoid organs. This finding suggests that the T cell response to therapy-induced lung damage may be mediated by specific tumor antigens, rather than being a result of nonspecific immune activation.

There is further evidence to support the notion of a clonal T cell selection within the tumor microenvironment (85). Specifically, in an analysis of BALF T cells from 10 patients with CIP, Suzuki et al. observed an increase in the number of PD-1^+^ and TIM-3^+^ CD8^+^ BALF T cells in patients with pneumonitis (86). Interestingly, CD8 cells in these patients also exhibited an increased expression of T cell immunoreceptor with immunoglobulin and ITIM domains (TIGIT). Notably, both TIGIT and TIM-3 are classified as second-wave immune checkpoints and are highly expressed in tumor infiltrating lymphocytes (87, 88). Collectively, these findings suggest that T cells present in the tumor microenvironment may relocate to other lung compartments following activation with checkpoint blocking antibodies, possibly localizing to certain coexpressed antigens (in both the tumor microenvironment and normal lung tissue) and ultimately leading to the development of pneumonitis. In fact, these cells may not be too dissimilar to the PD-1^+^ CD8^+^ T cells identified in our CIP cohort (68). The mechanisms described above could potentially account for the sporadic nature of CIP, as it relies on antigenic similarities between the tumor and lung parenchyma.

To this end, there have been several findings suggestive of molecular mimicry as a possible driver for CIP, particularly in the context of tumor mutational burden (TMB). Elevated TMB levels may be linked to a higher incidence of irAEs, potentially due to the development of neoantigens or the release of tumor antigens following cell death (89). These antigens can cause a cross-reaction with healthy tissue antigens, leading to the manifestation of irAEs. However, a recent meta-analysis failed to establish a significant correlation between TMB and irAE development (90), suggesting that other factors may also be at play. It is worth noting that a higher TMB was associated with increased tumor response rates, suggestive of a better immune trigger, though this did not necessarily translate into a higher incidence of toxicity (90). This highlights the complexity of the relationship between these two factors. Unraveling the association between the TMB and CIP may be attempted by identifying any overlap between the mutational burden in both CIP and cancer tissue samples with a corresponding T cell clone response within both compartments.

Most studies investigating CIP have focused on lymphocyte changes, leaving a gap in our understanding of myeloid cell alterations during the disease process. However, recent research has shed light on the involvement of proinflammatory macrophages in CIP. For example, in 37 patients with NSCLC, there was upregulation of proinflammatory macrophages in CIP identified using bulk RNA sequencing of surgical tissue specimens (91). In this research, macrophages from patients with CIP expressed higher levels of TNF and CXC chemokine ligand-10 (CXCL-10) compared with that in individuals in the control group (91). These findings align with previous flow cytometric analysis of CIP BALF specimens, which revealed distinct clusters of IL-1β^hi^, TNF-α^hi^, CD-11b^hi^ myeloid cells that were significantly upregulated in CIP BALF (68, 74), along with increased BALF protein levels of CXCL-10. Similarly, Franken et al. demonstrated comparable results regarding myeloid dysfunction in CIP; they identified two different clusters representing monocyte and macrophage cells (74). The first cluster consisted of IL-1β^hi^ monocytes with high expression of CXCL-10, while the second cluster had increased expression of proinflammatory macrophage genes, including IL-1β and TNF. Taken together, these findings may indicate that the observed changes in innate immune cells, specifically monocytes and macrophages with IL-1β^hi^TNF-α^hi^CXCL-10^hi^ profile, may play a central role in attracting lymphocytes to the alveoli, thus contributing to the pathogenesis of CIP.

### Upregulated levels of autoantibodies.

Humoral immunity may also play a role in the development of irAEs associated with ICI therapy. Autoantibodies, which may already be present at low levels prior to ICI therapy or produced de novo, have been implicated in the pathogenesis of some nonpulmonary irAEs. For example, in seven patients who developed bullous pemphigoid (an autoimmune blistering skin condition) after PD-L1 therapy, a unique antibody to a basement membrane protein was observed (92). Similarly, thyroid dysfunction has also been linked to ICI therapy, with many patients who developed thyroiditis having evidence of circulating antithyroid antibodies either present at baseline or developed during treatment (93).

With regards to autoantibodies in CIP, in a cohort of 66 patients experiencing irAEs, of which 14 (21%) developed pneumonitis, preexisting elevation of rheumatoid factor or antinuclear antibodies was significantly associated with irAE (94). Additionally, anti-CD74 autoantibodies have recently been implicated in patients with CIP (95). CD74 functions as a chaperone molecule involved in major histocompatibility complex II intracellular trafficking, and it acts as a high-affinity receptor for macrophage inhibitory factor, inducing inflammatory mediators and cell proliferation (96–98). High-throughput serological analysis of recombinant cDNA expression by Tahir et al. revealed a significant median 1.34-fold increase in anti-CD74 antibody levels after ICI treatment in CIP, while no significant changes were noted in a comparison group of 20 patients without pneumonitis (95). These findings suggest a potential role for antibody-mediated mechanisms in the development of CIP.

### Cytokine dysfunction.

Elevated levels of various cytokines have been associated with irAEs, including CIP. In fact, there are significant changes in cytokine levels in patients who develop irAEs following ICI treatment (99). Though clearly capable of contributing to lung injury, whether these cytokine increases are causally related to irAE development remains to be seen (33). Interestingly, some cytokines have shown potential as predictive biomarkers for irAEs. For example, in a discovery cohort of 58 patients with melanoma, samples taken at baseline and at the time of toxicity identified 11 signature cytokines (including granulocyte colony-stimulating factor, granulocyte macrophage colony-stimulating factor, fractalkine, basic fibroblast growth factor-2, IFN-α2, IL-12p70, IL-1a, IL-1β, IL-1 receptor agonist, IL-2, and IL-13) that strongly correlated with the development of severe irAEs, including two cases of pneumonitis (100). Another study looking specifically at 204 patients with NSCLC, of which 43 developed irAEs, found a similar proinflammatory increase in IL-1β cytokine but also elevations in IL-5, IL-8, IL-10, IL-12p70, and granzyme A and decreased G-CSF as predictors for irAEs that included pneumonitis (5 of a total of 43) (101). IL-5, IL-8, and IL-12p70 are considered proinflammatory cytokines (102–104). The actual mechanism as to how IL-5, a Th2 cytokine and a powerful eosinophil activator and recruiter (105), may be involved in lung injury of pneumonitis is uncertain. One plausible explanation may be in its secondary role of B cell stimulation and augmentation of immunoglobulin production (106), but how this may lead to developing CIP is unclear. IL-8 exerts its influence through a range of mechanisms, which encompass the enhancement of neutrophil activation, granule release, superoxide generation, and the expression of adhesion molecules (107). Additionally, receptors for IL-8 are present not only on neutrophils but also on Tregs, monocytes, and NK cells, indicating their potential involvement in the complex biology of CIP (108). Conversely, IL-10 is predominantly regarded as an antiinflammatory cytokine (109), but its role in autoimmune disease remains ambiguous, as illustrated by the failure of inducing an autoimmune syndrome in IL-10–deficient mice (109).

While one report suggested elevated IL-6 levels beyond baseline in CIP (110), a separate study of BALF cytokines in 12 patients diagnosed with CIP demonstrated significantly elevated IL-6 levels compared with those in individuals in the control group (111). However, it should be noted that IL-6 is not universally elevated in CIP BALF (68). Yet, tocilizumab, an IL-6 inhibitor, has been shown to be effective in treatment of steroid refractory CIP in a single-center experience report (112). In a separate analysis of serum and BALF of 13 patients with CIP after PD-1/PD-L1 therapy for NSCLC, elevation in both IL-17A and IL-35 was observed in both compartments (113). Furthermore, serum IL-17A levels were found to positively correlate with the Th17 cellular subtype. IL-17A has been implicated in other autoimmune disorders (114), acute lung injury (115), and lung fibrosis (116), which implies that it may also contribute to the pathogenesis of CIP. A summary of cytokines that have been shown to be deranged in CIP are outlined in Table 3. Although the underlying tumor histology, host factors, and disease profiles vary among patients, there is a growing body of evidence to suggest that cytokine dysregulation plays a role in the pathogenesis of pneumonitis. While a distinct cytokine signature has yet to be identified due to these differences, this may hint at the presence of multiple pathways at play in pneumonitis.

### Genetic predisposition.

As the development of irAEs are widely believed to be associated with autoimmunity, genetic variations have been investigated as a potential contributing factor. Various genes with single nucleotide polymorphisms have been linked to different irAEs (117–119), indicating a complex interplay of multiple pathways. Of particular interest are the human leukocyte antigen (HLA) variations, as they are critical in the immune cell interface. In a cohort of 256 patients receiving ICI treatment, including 29 cases of CIP, HLA typing demonstrated a strong correlation between CIP rates and germline expression of *HLA-B* allele 35 and *HLA-DRB1* allele 11 (120). These genes are also associated with other autoimmune disorders (118, 121, 122), highlighting the possible role of genetic factors in the pathogenesis of CIP. In more recent work, investigators looked at the T cell receptor β variable (TRBV) in the peripheral blood leukocytes of 81 individuals with different malignancies (123). Interestingly, they uncovered a certain *TRBV* allele haplotype that either reduced or increased the risk of severe (≥grade 3) irAEs. While *TRBV* polymorphism has been linked to autoimmune diseases (124), its association with irAEs has not been demonstrated previously (125).

IL-7 is a critical cytokine for lymphocyte homeostasis, and it has been shown to regulate the number of circulating T cells in humans (126). In a genome-wide association study of 1,751 patients on ICIs across multiple cancer types, several significant single nucleotide polymorphisms near IL-7 were identified that associated with ICI toxicity in general (127). These germline variants of IL-7 demonstrated higher lymphocyte stability after ICI initiation that consequently increased the risk of irAEs (127). Although not yet fully understood, these findings suggest that genetic variations likely contribute to the dysregulation leading to CIP.

### The microbiome.

Extensive work has investigated the gut microbiome where certain flora dictate both response and ICI-induced colitis rates (128). Abundance of *Bacteroidetes* phylum has been shown to be protective against the development of ICI-induced colitis in a cohort (129), whereas another group demonstrated that *Faecalibacterium*-enriched gut microbiota was associated with more frequent ICI-induced colitis (130). The increase in colitis risk in these patients was mediated by the upregulation of T cell response through higher inducible T cell costimulator induction thought to be mediated by increased circulating IL-2 after immunotherapy. A similar process may be occurring in the lungs, but the bulk of the microbiome studies related to ICI response have focused on the gut, and much of the lung microbiome work has focused on the risk of lung cancer development (131), highlighting this as an area in need of further investigation.

## Potential targets for future research

It is worth noting that the absence of a clear link between CIP and specific biological characteristics could be due to limitations in research studies or disease-related factors. The incidence of CIP may be too low in some cancers to identify any meaningful associations. Additionally, the misclassification of pulmonary disease as CIP could confound results. Furthermore, the immune response is complex, and multiple mechanisms may be present across different patient populations. An important variable that may lead to distinct pathobiological pathways in CIP may be the underlying tumor histology. For instance, patients with NSCLC have a higher risk of CIP than other malignancies (58, 59). This phenomenon may be dictated by tumor immunobiology that is often disparate.

To gain a better understanding of the pathogenesis of CIP, several potential avenues can be explored. First, it is necessary to understand how checkpoint blockade affects the immune landscape and function within the lung in the absence of any pathological processes. Therefore, conducting studies to investigate the effect of ICI alone on immune cell subsets would be instructive. The recent interest in use of ICIs in earlier stage cancers and nonmalignant disease presents an opportunity to study the effect of ICI in conditions in which the tumor burden is either very low or absent (132, 133). In addition, interrogating the lung microbiome could provide crucial data about potential disruptions that might contribute to CIP.

An effective approach to gain further insight into the pathogenesis of CIP would be to develop an animal model of ICI pneumonitis. Several models have been attempted, but a robust prototype is still lacking. Gao et al. used humanized mice treated with collagen-specific antibodies, followed by immune checkpoint blockade drugs leading to development of arthritis and pneumonitis (134). These mice showed increased inflammation in lung tissue and elevated levels of TNF^+^ CD4- and CD8-infiltrating T cells in the lungs and peripheral blood. Another murine model used dual checkpoint (PD-1 and CTLA-4) blockade, causing lung inflammation with systemic T cell activation, suggesting that immunotherapy-mediated peripheral activation of T cells may be the initial immune derangement leading to CIP (135). However, the model lacked specificity due to concerns of high-dose checkpoint blockade, multiorgan irAE involvement, and genitourinary developmental abnormalities in the mice used. Nonetheless, a more stringent CIP model remains important, as it could enable the evaluation of temporal changes in the clinical course of the disease that are difficult to capture in real-world cases. This would also allow a more detailed and thorough analysis of biological changes that are difficult to yield in human studies.

There is another limitation that hampers progress in this field, which pertains to our incomplete understanding of the in vivo pharmacokinetics of ICIs and their true half-life. When examining patients with ICI-induced myocarditis, it was observed that levels of ICI drugs remained elevated for several months (136). Moreover, despite receiving plasmapheresis and steroid treatment, these patients experienced prolonged suppression of PD-1 detection on T cells, as measured by flow cytometry. Additionally, significant variation was noted in the duration of ICI effects among different patients, further complicating our comprehension of the intricate interactions between ICIs and lymphocytes (136). Further research of in vivo ICI pharmacokinetics is therefore warranted.

In summary, checkpoint inhibitor therapy has revolutionized the landscape of cancer treatment. However, its use has been limited by related immune toxicities, especially CIP. The pathogenesis of CIP is marked by an elevated T cell–mediated immune activation, disruption in cytokine balance, autoantibody upregulation, and underlying genetic predispositions, all contributing to lung inflammation. Certain T cell subsets appear to have greater prominence within the disease context, potentially playing pivotal roles in CIP. Despite these observations, many questions regarding the pathophysiology of CIP remain unanswered. Hence, it is imperative to undertake additional research centered on comprehending the immune landscape and establishing a robust disease model. This approach is essential to enhance our insight into CIP and formulate efficacious treatment strategies.

## Figures and Tables

**Table 3:**
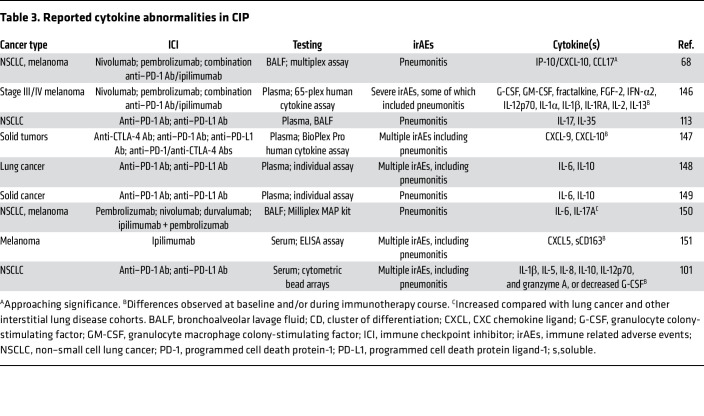
Reported cytokine abnormalities in CIP

**Table 2:**
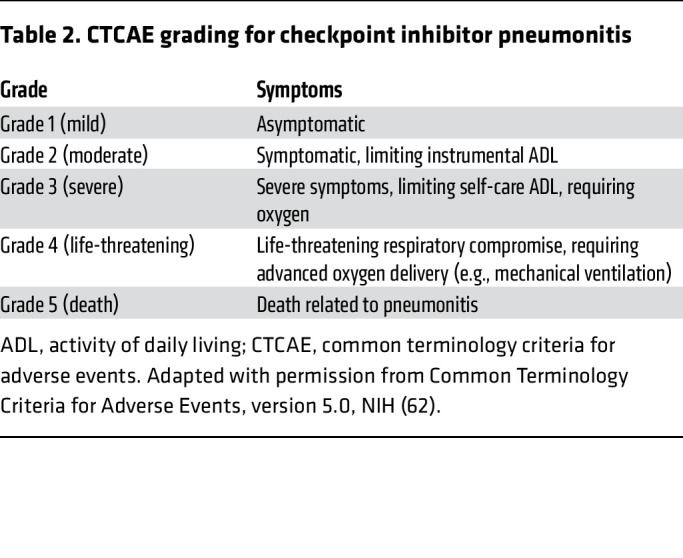
CTCAE grading for checkpoint inhibitor pneumonitis

**Table 1:**
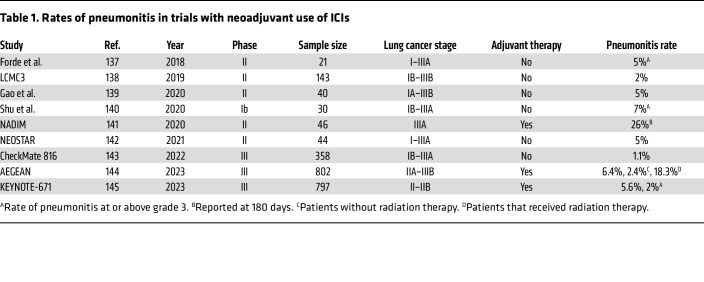
Rates of pneumonitis in trials with neoadjuvant use of ICIs

## Version 1 01/16/2024

Electronic publication
